# Effects and safety of traditional Chinese medicine on the gut microbiota of an adult with chronic kidney disease: A protocol for systematic review and meta-analysis

**DOI:** 10.1097/MD.0000000000028847

**Published:** 2022-02-18

**Authors:** Li Huang, Xin Luo, Ming Chen

**Affiliations:** a School of Clinical Medicine, Chengdu University of Traditional Chinese Medicine, Chengdu, PR China; b Department of Nephropathy, Hospital of Chengdu University of Traditional Chinese Medicine, Chengdu, PR China.

**Keywords:** chronic kidney disease, gut microbiota, meta-analysis, traditional Chinese medicine

## Abstract

**Background:**

Studies have reported that changes in the abundance of gut microbiota may explain the medically helpful responses towards traditional Chinese medicine (TCM), an essential part of alternative and complementary medicine, in treating chronic kidney disease (CKD). This study aimed to illuminate the changes in the abundance of gut microbiota of TCM with CKD.

**Methods:**

The meta-analysis was performed in the PubMed, Web of Science, EMBASE CNKI, WanFang, VIP, and CBM databases, from its inception to October 2021 to discriminate randomized controlled trials and examine the effects of TCM on CKD. Two researchers independently performed literature selection, data extraction, and quality assessment. The risk of bias of the included studies was estimated by taking into consideration the characteristics including random sequence generation, allocation concealment, blinding of patients, blinding of outcome assessment, completeness of outcome data, selective reporting, and other bias using Cochrane Collaboration's tool for assessing the risk of bias.

**Results and Conclusions:**

From the perspective of intestinal flora, this study will provide high-quality evidence for the effectiveness and safety of TCM for CKD. It provides a new therapeutic approach for treating CKD with Chinese herbal medicine combined with Western medicine.

**Inplasy registration number:**

INPLASY2021100118.

## 1 Introduction

Chronic kidney disease (CKD) is one of the major diseases that endanger human health. With the aggravation of population aging and the increasing incidence of diabetes and hypertension, the prevalence of CKD is increasing year by year.^[1]^ According to statistics, the prevalence of CKD is about 8% to 16% worldwide,^[2]^ and the growth rate of all-cause mortality is the sixth in the world, and the first in China.^[^3^,^^4^^]^ CKD is an important cause of cardiovascular disease, all-cause death, and people's quality of life decline. In addition, CKD also brings heavy economic burden to the country and society,^[5]^ so it is urgent to delay the progress of CKD.

Treatment of CKD from the perspective of gut microbiota is attracting more and more attention. Intestinal microecology and the human body exhibit mutually beneficial relationships in a dynamic balance. Nevertheless, due to gut-derived uremic toxins in CKD patients, the abundance and structure of gut microbiota are damaged. In turn, the dysbiosis of the gut microbiota contributes to circulating uremic toxins, systemic inflammation, accelerating the development or increasing of CKD, and all-cause deaths.^[6]^ The imbalance in the intestinal flora in the human body leads to increased protein-binding poisonous chemicals such as p-cresyl sulphate and indoxyl sulphate (IS). These toxic chemicals are implicated in oxidative stress, inflammation, uremia, worsening of kidney-related function. Thus, ultimately increasing the danger of cardiovascular events and death.^[7]^

Currently, the management of gut microbiota in patients with CKD mostly depends on adjusting patients’ diet and supplementing with prebiotics and probiotics. Chauveau et al^[8]^ reported that the beneficial effects of the Mediterranean diet on endothelial function, inflammation, lipid profile, and blood pressure of patients with CKD reduced kidney-related decline and improved survival. March et al^[9]^ reported that prebiotic, probiotic, and symbiotic supplements substantially affect enteric-received poisonous metabolites, lipid profiles, and clinical outcomes in patients undergoing hemodialysis or peritoneal dialysis. However, it still cannot meet the needs of patients and solve the clinical needs, so it is urgent to seek new clinical treatment. Traditional Chinese medicine (TCM) regulates gut microbiota has become the one of research hotspots of CKD. Chinese herbal medicine (CHM) is the main component of TCM, many studies have confirmed that CHM combined with Western medicine can significantly increase the abundance of *bifidobacteria*, reduce *Colibacillus* and Enterococcus, reduce the levels of IS, endotoxin and lipopolysaccharide, regulate intestinal flora, and delay the process of CKD.^[^10^,^^11^^]^

However, no meta-analysis has reported the effect of TCM on the gut microbiota of patients with CKD. In line with this, we provide a protocol to evaluate the results of randomized controlled trials (RCTs) to examine the benefits of TCM on controlling gut microbiota.

## 2 Method

### 2.1 Design

Meta-analysis will use the protocol designed according to the Preferred Reporting Project Guidelines for systematic review and meta-analysis protocol (PRISMA-P).^[12]^

### 2.2 Protocol registration

The protocol has been registered on INPLASY.COM (registration number: INPLASY2021100118).

### 2.3 Inclusion criteria

#### 2.3.1 Population

Adult patients (aged >18 years) diagnosed with CKD, including those who underwent hemodialysis, peritoneal dialysis, or did not undergo dialysis, regardless of gender, race, and area.

#### 2.3.2 Interventions

CHM interventions in the experimental group in the included studies consisted of taking measures of Chinese medicine enema, oral Chinese medicine, such as Chinese herbal compounds, Chinese patent medicine, and single Chinese medical herbs, which can be administered in the form of decoctions, granules, or powders.

#### 2.3.3 Control

The control group included individuals treated with either Western medicine or placebo, or those who were treated with either Western medicine or placebo did not receive any intervention.

#### 2.3.4 Outcomes

Gut microbiota as well as its metabolites (fecal metabolome and ribosomal RNA sequencing), including, but not limited to the changes in the following measures indicators.

1. *Colibacillus*, *Enterococcus*, *Bifidobacterium*, *Lactobacillus*, D-lactate, endotoxin, p-cresyl sulphate, IS, trimethylamine-*N*-oxide as the main outcomes.
2. Renal function such as estimated glomerular filtration rate, serum creatinine, blood urea nitrogen, was secondary outcomes. Finally, meta-analysis was conducted by RevMan software (San Francisco, USA).

Besides, a doubling of serum creatinine, 30% decrease in estimated glomerular filtration rate, electrolyte markers (serum potassium ≥5.5 mmol L^−1^) and the adverse events will be included to evaluate the safety of TCM intervention.

### 2.4 Exclusion criteria

The following were the exclusion criteria: study design: non-RCTs, such as retrospective studies, observational studies, case reports, and cross-over studies; studies that could not be used for statistical analysis due to incomplete data; repeated data studies; review.

### 2.5 Search strategy

#### 2.5.1 Information databases

Using electronic databases (Web of Science, PubMed, CNKI, Wanfang Database, CBM, and VIP Database) to search by titles and abstracts of TCM for the gut microbiota of adult patients with CKD, were searched up to October 30, 2021, as restricted to English and Chinese.

#### 2.5.2 Search terms

The following terms were explored: (“chronic renal failure” or “chronic kidney disease” or “chronic renal insufficiency” or “CKD” or “CRF”) and (“gut microbiota” or “gastrointestinal microbiome” or “Intestinal Microbiota” or “gut flora” or “dysbiosis” or “Bacteria, Enteric”) and (“traditional Chinese medicine” or “TCM” or “Chinese herbal medicine” or “Chinese medicine enema” or (“Chinese” and (“medicine” or “decoction” or “formula” or “prescription” or “granule”)) or “Chinese herbal compound prescription”) and (“randomised controlled trial” or “controlled clinical trial” or “RCT”).

### 2.6 Study selection

Two researchers independently compiled articles in databases based on the inclusion mentioned above and exclusion criteria. Studies that are not RCT and do not contain CKD will be excluded by reading title and abstract. And extracted the following data and entered into data extraction tables: basic characteristics (e.g., title, first author's name, publication date, intervention schedule of treatment, control group, and treatment duration), participant characteristics (e.g., age and sample size), outcome measures, and adverse events. If they failed to reach a consensus, a third researcher was consulted.

### 2.7 Data extraction

Data will be extracted from the eligible studies by 2 authors independently with same pre-designed data extraction table. And extracted the following data and entered into data extraction tables: basic characteristics (e.g., title, first author's name, publication date, intervention schedule of treatment, control group, and treatment duration), participant characteristics (e.g., age and sample size), outcome measures, and adverse events.

### 2.8 Risk assessment of bias

The methodological quality and risk of bias (ROB) of all included studies were assessed by 2 authors independently using the Cochrane Collaboration's tool. The ROB in randomization, allocation, and loss of follow-up of the included RCTs was evaluated according to the systematic review of interventions in The Cochrane Manual 5.3.

### 2.9 Statistical analysis

Review Manager, a systematic review software designed by the Cochrane Collaboration, was used for data analysis. The mean and standard deviation of each study were computed and pooled as mean difference or standardized mean differences (SMD) with a 95% confidence interval (CI) considering the diversity of interventions and potential heterogeneity among the included studies and to determine heterogeneity (*P* ≥ .05 or *I* ^2^ ≤ 50% = low heterogeneity, fixed effect model; *P* < .0.5 or *I* ^2^ > 50% = random effect model, high heterogeneity, or subgroup analysis). The quality of evidence will be performed by the grades of recommendation, assessment, development and evaluation.^[13]^

## 3 Discussion

TCM is one of the primary, complementary, and alternative medicines licensed worldwide and has thousands of years of history in China. Because of its distinctive theoretical system, TCM is mainly popular in Asian countries. In clinical practice, TCM, along with its unique theory based on holistic treatment and syndrome differentiation, has positive effects in patients with CKD, and evidence for the same has been obtained from several RCT over the past few years.^[^14^,^^15^^]^ TCM is attracting more and more attention around the world as a complementary and alternative therapy.

In recent years, researchers have proposed the therapeutic mechanism of TCM against CKD from the view point of gut microbiota, and evidence indicates that regulating the gut microbiota could positively affect patients with CKD.^[^16^,^^17^^]^ We are trying to select more clinical trials and use meta-analysis to provide scientific and reliable clinical basis for the efficacy and safety of CHM in the treatment of CKD from the perspective of intestinal flora, so as to provide a basis for clinical treatment programs with fewer side effects and high efficacy.

## Author contributions

**Conceptualization:** Li Huang.

**Data curation:** Li Huang, Xin Luo.

**Formal analysis:** Li Huang, Xin Luo.

**Funding acquisition:** Ming Chen.

**Investigation:** Ming Chen.

**Methodology:** Li Huang, Xin Luo.

**Project administration:** Ming Chen.

**Resources:** Ming Chen.

**Software:** Li Huang, Xin Luo.

**Supervision:** Ming Chen.

**Validation:** Ming Chen.

**Visualization:** Li Huang.

**Writing – original draft:** Li Huang.

**Writing – review & editing:** Ming Chen.

## References

[1] Vos T Lim SS Abbafati C. Global burden of 369 diseases and injuries in 204 countries and territories, 1990-2019: a systematic analysis for the Global Burden of Disease Study 2019. Lancet 2020;396:1204–22. 33069326 10.1016/S0140-6736(20)30925-9 PMC7567026

[2] Wang F He K Wang J. Prevalence and risk factors for CKD: a comparison between the adult populations in China and the United States. Kidney Int Rep 2018;3:1135–43. 30197980 10.1016/j.ekir.2018.05.011 PMC6127437

[3] Rahman M Xie D Feldman HI. Association between chronic kidney disease progression and cardiovascular disease: results from the CRIC study. Am J Nephrol 2014;40:399–407. 25401485 10.1159/000368915 PMC4275411

[4] Palit S Kendrick J. Vascular calcification in chronic kidney disease: role of disordered mineral metabolism. Curr Pharm Des 2014;20:5829–33. 24533939 10.2174/1381612820666140212194926 PMC4130905

[5] Leaf IA Duffield JS. What can target kidney fibrosis? Nephrol Dials Transplant 2017;32: (suppl): i89–97. 10.1093/ndt/gfw388 28391346

[6] Lynch SV Pedersen O. The human intestinal microbiome in health and disease. N Engl J Med 2016;375:2369–79. 27974040 10.1056/NEJMra1600266

[7] Meijers B Evenepoel P Anders H-J. Intestinal microbiome and fitness in kidney disease. Nat Rev Nephrol 2019;15:531–45. 31243394 10.1038/s41581-019-0172-1

[8] Chauveau P Aparicio M Bellizzi V. Mediterranean diet as the diet of choice for patients with chronic kidney disease. Nephrol Dial Transplant 2018;33:725–35. 29106612 10.1093/ndt/gfx085

[9] March DS Jones AW Bishop NC Burton JO. The efficacy of prebiotic, probiotic, and synbiotic supplementation in modulating gut-derived circulatory particles associated with cardiovascular disease in individuals receiving dialysis: a systematic review and meta-analysis of randomized controlled trials. J Renal Nutr 2020;30:347–59. 10.1053/j.jrn.2019.07.006 31607550

[10] Chen XJ Gao SH Ruan MN. Shen-Shuai-Ning granule decreased serum concentrations of indoxyl sulphate in uremic patients undergoing peritoneal dialysis. Biosci Rep 2018;38:01–8. 10.1042/BSR20171694 PMC6137246 29921575

[11] Zheng L Chen S Wang F. Distinct responses of gut microbiota to Jian-Pi-Yi-Shen decoction are associated with improved clinical outcomes in 5/6 nephrectomized rats. Front Pharmacol 2020;11:604–15. 32435197 10.3389/fphar.2020.00604 PMC7219274

[12] Moher D Shamseer L Clarke M. Preferred reporting items for systematic review and meta-analysis protocols (PRISMA-P) 2015 statement. Syst Rev 2015;4:01–9. 10.1186/2046-4053-4-1 PMC4320440 25554246

[13] Higgins JPT Altman DG Gøtzsche PC. The Cochrane Collaboration's tool for assessing risk of bias in randomised trials. BMJ 2011;343:01–9. 10.1136/bmj.d5928 PMC3196245 22008217

[14] Li S Yin XX Su T Cao C Li X Rao XR. Therapeutic effect of Astragalus and Angelica mixture on the renal function and TCM syndrome factors in treating stage 3 and 4 chronic kidney disease patients. Chin J Integr Tradit Western Med 2014;34:780–5. 25137839

[15] Wang YJ He LQ Sun W. Optimized project of traditional Chinese medicine in treating chronic kidney disease stage 3: a multicenter double-blinded randomized controlled trial. J Ethnopharmacol 2012;139:757–64. 22178174 10.1016/j.jep.2011.12.009

[16] Wang Y Li J Chen C. Targeting the gut microbial metabolic pathway with small molecules decreases uremic toxin production. Gut Microbes 2020;12:01–19. 10.1080/19490976.2020.1823800 PMC7577114 33016221

[17] Ji CL Deng YS Yang AC. Rhubarb enema improved colon mucosal barrier injury in 5/6 nephrectomy rats may associate with gut microbiota modification. Front Pharmacol 2020;11:01–14. 10.3389/fphar.2020.01092 PMC7403201 32848732

